# RNA-seq dataset of the chorioallantoic membrane of male and female chicken embryos, after 11 and 15 days of incubation

**DOI:** 10.1016/j.dib.2024.110830

**Published:** 2024-08-14

**Authors:** Christelle Hennequet-Antier, Maeva Halgrain, Sophie Réhault-Godbert

**Affiliations:** a Université Paris-Saclay, INRAE, MaIAGE, 78350 Jouy-en-Josas, France; b Université Paris-Saclay, INRAE, BioinfOmics, MIGALE Bioinformatics Facility, Jouy-en-Josas, France; c INRAE, Université de Tours, BOA, 37380 Nouzilly, France

**Keywords:** Bird, Extra-embryonic membrane, Developmental stage, Sex, Transcriptome, Differentially expressed genes, Gene ontology

## Abstract

The chicken chorioallantoic membrane (CAM) is an extraembryonic structure that exhibits many vital functions to support the development of the chicken embryo (gaseous exchange, innate defence, calcium transport from the eggshell to the embryo skeleton, homeostasis). Developing from day 6 of incubation, the CAM progressively differentiates into three functional layers (the chorionic epithelium in contact with the inner eggshell, the highly vascularized mesoderm, and the allantoic epithelium), between 11 and 15 days of incubation. This article describes the RNASeq dataset and the analyses performed on total CAMs collected from male and female embryos after 11 and 15 days of incubation. The datasets are available at the NCBI Gene Expression Omnibus (GEO) repository (http://www.ncbi.nlm.nih.gov/geo) using GSE199780 as the accession number. The statistical analysis of the data allowed identifying genes differentially expressed depending on the sex of the embryo at two time points of CAM differentiation. Knowing that the CAM is widely used as a model to study tumour growth, metastasis or wound healing, the resulting analysis highlights the necessity to include this sex variable in experimental assays to avoid any bias of interpretation. Indeed, the functional annotation of genes that are differentially expressed between male and female CAMs revealed an enrichment of activities and functions related to lipid metabolism, bone formation, and morphogenesis suggesting that the response of the CAM to external and experimental stimuli might be different depending on the sex of the embryo.

Specifications Table

**Table:** 

Subject	Transcriptomics
Specific subject area	RNAseq dataset of the chicken chorioallantoic membrane between males and females after 11 and 15 days of incubation
Data format	Raw, Filtered, Analyzed
Type of data	Table, Graph, Figure
Data collection	Total RNA was extracted from chicken chorioallantoic membranes collected from male and female embryos after 11 and 15 days of incubation (*n* = 40), using Nucleospin RNA kit. The sample quality was checked (260 nm/280 nm, migration of 1 % agarose gel and using Agilent 2100 bioanalyzer), and submitted to RNA sequencing ((Illumina Novaseq 6000). To identify differentially expressed genes between incubation day 15 and incubation day 11, and between CAM from males and CAM from females, statistical analyses were performed on raw data using EdgeR.
Data source location	Institut National de Recherche pour l'Agriculture, l'Alimentation et l'Environnement (INRAE) Nouzilly, Région Centre Val de Loire France Latitude N 47° 32′ 41.046″ *Longitude E 0° 46′ 54.595*
Data Accessibility	Repository name: NCBI Gene Expression Omnibus (GEO) repository (http://www.ncbi.nlm.nih.gov/geo) Data identification number: GSE199780 as the accession number Direct URL to data: https://www.ncbi.nlm.nih.gov/geo/query/acc.cgi?acc=GSE199780 The script R and the results for the differential analysis and the functional enrichment tests (supplementary files Tables S1-S3) were stored in the repository Hennequet-Antier, Christelle, 2024, “CAM_RNA-Seq”, https://doi.org/10.57745/6YDAQD, Recherche Data Gouv.
Related research article	M. Halgrain, N. Bernardet, C. Hennequet-Antier, S. Réhault-Godbert, Sex-specific transcriptome of the chicken chorioallantoic membrane, Genomics 116 (2024) pp.110754. https://doi.org/10.1016/j.ygeno.2023.110754 https://hal.inrae.fr/hal-04356368v1

## 1 Value of the Data

• The chorioallantoic membrane is a complex tissue involved in many vital functions for the developing avian embryo, and as such, is used as an experimental model in various fields of biological research.
• The dataset provides a full list of genes of which expression differs between CAM from male and female embryos, bearing in mind that differences due to the effect of sex are rarely taken into account when investigating extra-embryonic structures.
• This article includes statistical analyses and functional enrichment of differentially expressed genes.
• The dataset may be used as a reference transcriptome of the chicken CAM by animal physiologists working in the field of developmental biology and sexual dimorphism, as well as by scientists using the CAM tissue for research on vascular study, cancer, drug screening, and development.

## 2 Background

The RNAseq dataset contains the gene expression in the chorioallantoic membrane (CAM) of chicken male and female embryos, between 11 and 15 days of incubation, which corresponds to the immature CAM (not differentiated) and the active CAM (fully differentiated), respectively. This dataset supports the discussion of two published articles [1,2]. In avian eggs, the chorioallantoic membrane is a highly vascularized structure that develops onto the inner part of the eggshell [3]. It ensures multiple physiological functions to accompany the development of the embryo [4,5]. Most studies on chicken CAM focus on its use as an in vivo and in vitro model for cancer and toxicology experiments [6]. In contrast, fundamental knowledge of the physiology of the CAM and the molecular players associated with its functions remain poorly documented. The present data article focuses specifically on the sex-linked expression of CAM [2] and provides a functional enrichment of the genes expressed differently according to sex. These RNAseq dataset were recently used for the development of a user-friendly tool to explore the expression of lncRNA and protein-coding genes (GRCg7b chicken assembly), in the CAM and 46 other chicken tissues [7].

## 3 Data Description

The CAM samples RNAseq data are available at NCBI Gene Expression Omnibus (GEO) repository (http://www.ncbi.nlm.nih.gov/geo) with GSE199780 as the accession number. Genes involved at CAM differentiation over days of incubation (after either 11 or 15 days, EID11 or EID15, respectively) were discussed in [1]. In the present work, we investigated gene expression data in relation to the sex difference in CAM and to provide additional data (functional enrichment) to the article [2]. Two lists of genes that are differentially expressed between male and female CAM were obtained for each embryonic incubation day, EID11 and EID15.

**Fig. 1:**
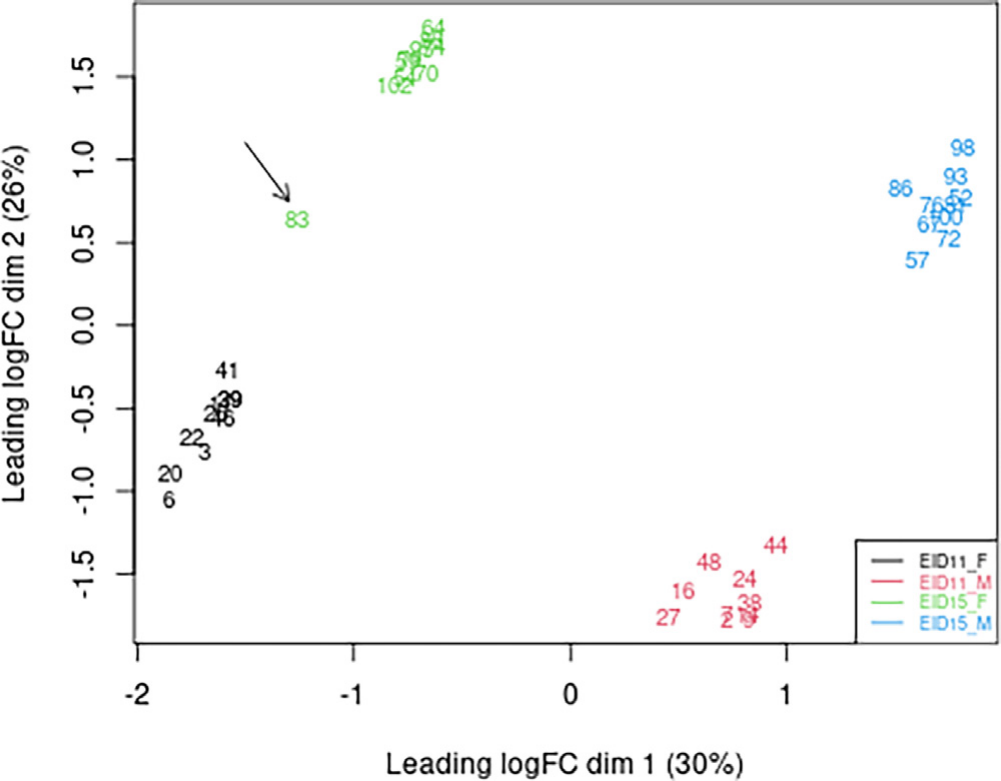
Multidimensional scaling (MDS) plot corresponding to RNAseq data from CAM collected from male and female embryos after 11 and 15 days of incubation. EID11_F: CAM collected from female embryos after 11 days of incubation (black); EID11_M: CAM collected from male embryos after 11 days of incubation (red); EID15_F: CAM collected from female embryos after 15 days of incubation (green); EID15_M: CAM collected from male embryos after 11 days of incubation (blue).(For interpretation of the references to color in this figure legend, the reader is referred to the web version of this article.)

Fig. 1 shows the Principal Component Analysis performed on the whole dataset. The top 500 genes are used to calculate the distance between expression profiles of samples. The distance approximates the log2 fold change between the samples. The results clearly indicate that all four groups (EID11_male, EID11_female, EID15_male, and EID15_Female) are distinct, with the exception of EID15_female sample number 83 that was removed from the subsequent statistical analyses. The first and second axes separate female CAMs from male CAMs, and the EID11 stage from the EID15 stage, respectively.

**Fig. 2:**
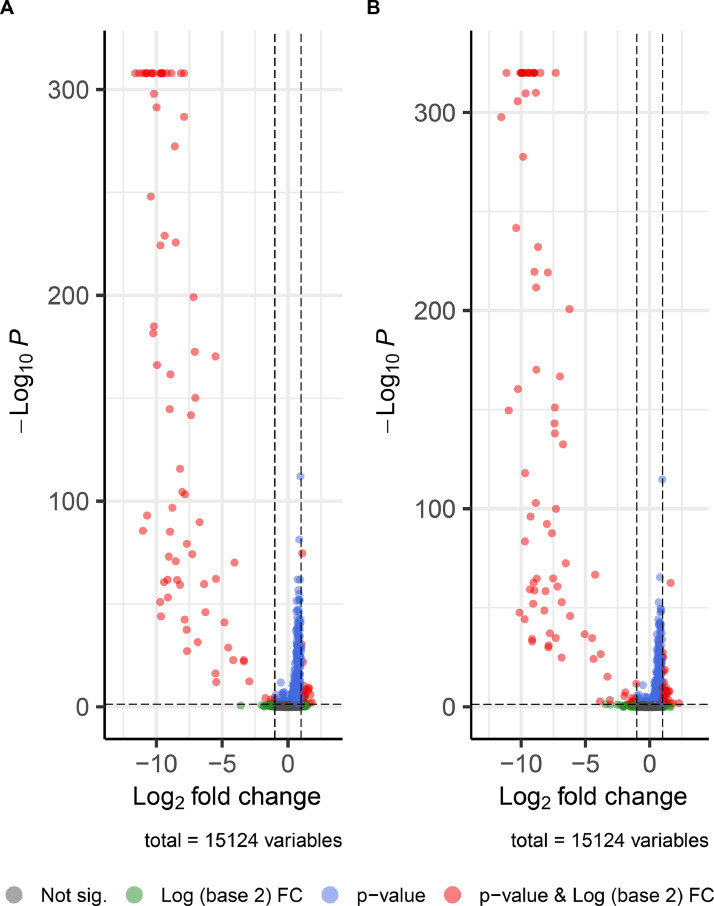
Volcano plots of CAM RNAseq for EID11 (A) and EID15 (B) between males and females. The scatterplot showing statistical significance (adjusted *P*-values) versus fold change (log2FC) corresponding to the magnitude of the difference for EID11 and EID15 are shown in A and B, respectively. Volcano plots were obtained using the Enhanced Volcano package (version 1.18.0). For both analyses, the cut-off for log2FC is >|1| and the cut-off for adjusted *P*-value is 0.05. Genes overexpressed in female CAM are shown of the left side of the scattered line while the genes that are overexpressed in the male CAM are shown on the right side.

For each gene, raw data, normalized data and results of the differential analysis between male and female CAM within EID11 and EID15 are presented in supplementary files (Table S1, Table S2). In brief, 15,124 genes are expressed in both EID11 and EID15, including XLOC genes. There are almost three times as many up-regulated genes (more expressed in males) as down-regulated genes (more expressed in females): 442/157 and 439/134 genes that are up/down regulated for the statistical test male versus female within EID11 and EID15, respectively. The resulting Volcano plots are shown in Fig. 2.

**Fig. 3:**
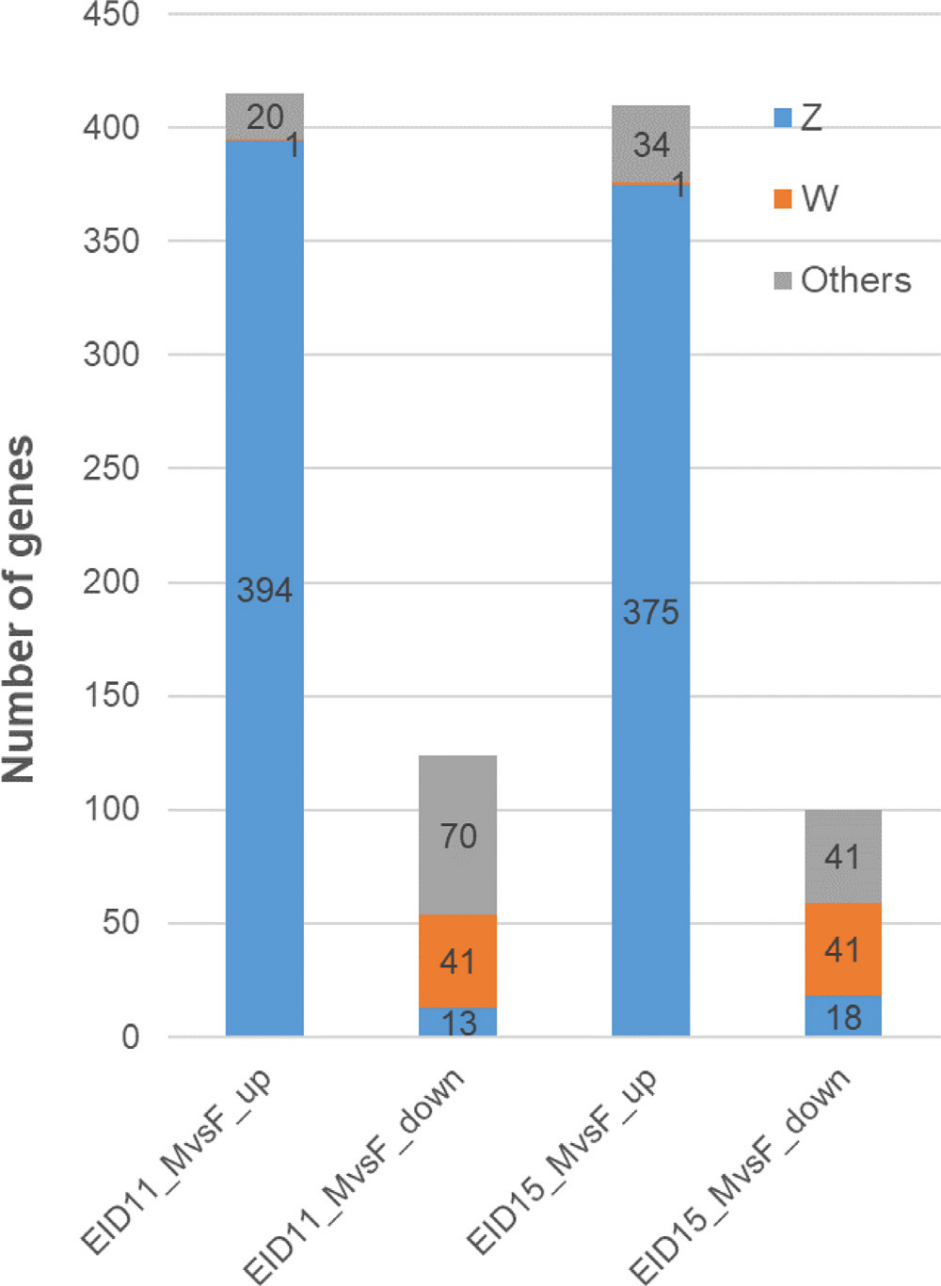
Chromosomal location of sex-differentially expressed genes (Sex-DEG) in CAMs after 11 (EID11) and 15 days (EID15) of incubation. Blue: SexDEG located on chromosome Z; Orange; SexDEG located on chromosome W; Grey: sex-DEG located on autosomes. One gene overexpressed in male has been assigned to chromosome W (ENSGALG00000047434). This discrepancy is likely due to errors in GRCg6a (GCA_000002315.5) assembly as it is no longer in the database.(For interpretation of the references to color in this figure legend, the reader is referred to the web version of this article.)

Not surprisingly, most of the differential genes between male and female CAMS are located on the W and Z sex chromosomes (Fig. 3).

**Fig. 4:**
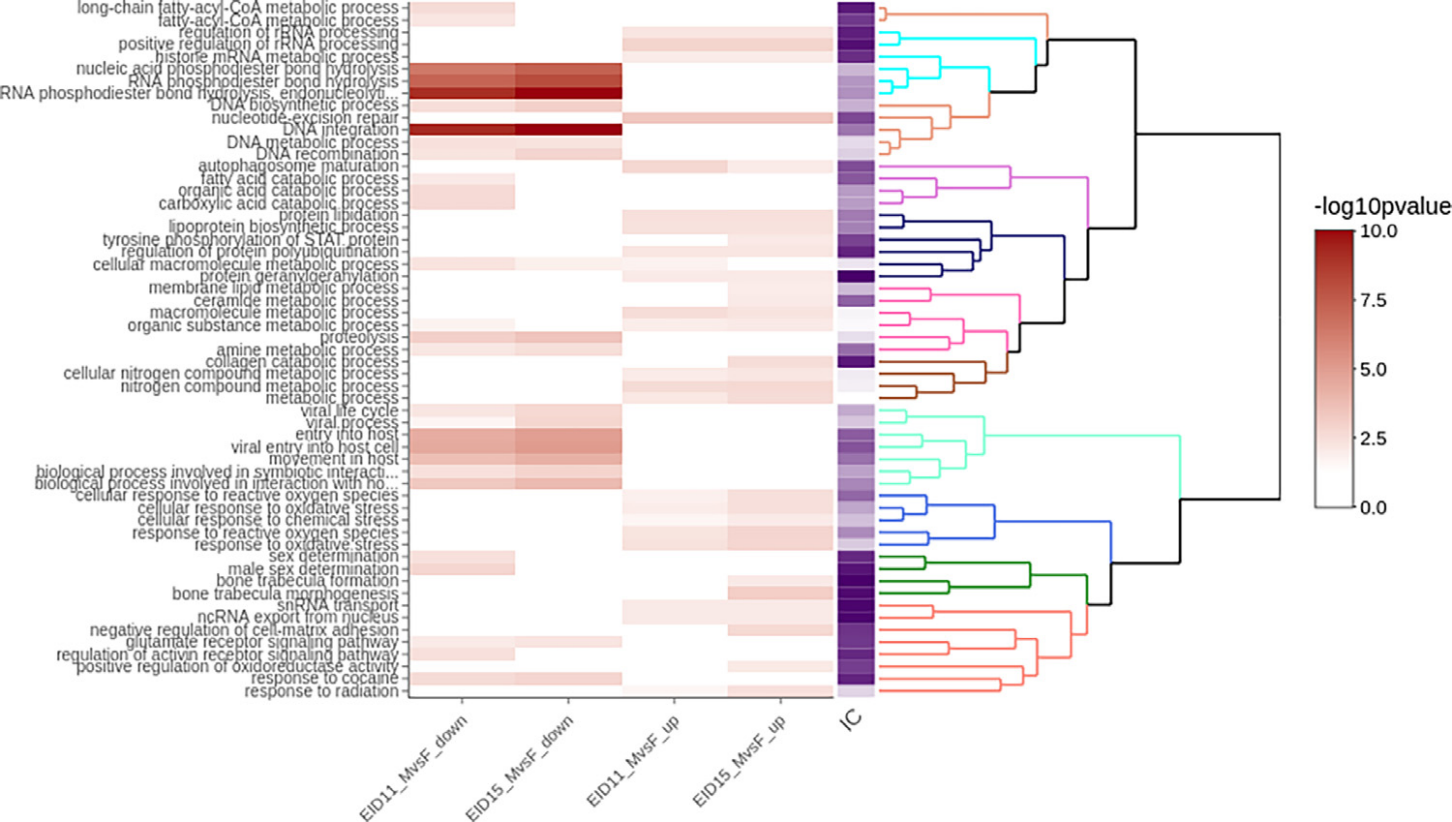
Hierarchical clustering algorithm of the four lists of enriched GO terms. The dendrogram is produced by a hierarchical clustering algorithm based on the Wang's distance between GO terms and the ward.D2 aggregation criteria. Clusters with similar semantic similarity between the GO terms were identified after a dynamic cutting and the branches of the dendrogram are colored. The heatmap shows the -log10 (*p*-value) of the enrichment test of the four lists of GO terms. The information content of each GO term is also visualized. IC, information content that is computed as the negative log probability of occurrence of the term within all GO terms.

The data were further analyzed to provide functional annotation of the genes based on Gene Ontology (GO). Functional enrichment tests were performed using over-representation analyses of the four lists of differentially expressed genes compared to the background of expressed genes. The Supplementary file (Table S3) shows the association between differentially expressed genes and GO annotated biological processes that were significantly enriched. The enriched GO terms related to biological processes for each list (26 terms in EID11_MvsF_down, 18 terms in EID15_MvsF_down, 17 terms in EID11_MvsF_up and 30 terms in EID15_MvsF_up) are grouped into 57 unique enriched GO terms for the four lists. These enriched GO terms were organized into 10 clusters with similar semantic similarity using a hierarchical clustering algorithm based on the Wang's distance between GO terms and the ward.D2 aggregation criterion (Fig. 4). This interactive figure includes a dendrogram, a heatmap of the functional enrichment tests and the information content (IC) of each GO term.

From this analysis, we highlighted many GO terms associated with lipid metabolism including long-chain fatty-acyl-CoA metabolic process (GO:0035336, cluster 1, IC = 8.83) with two genes down-regulated at EID11 (ELOVL7 located on Z chromosome, and DGAT2), fatty-acyl-CoA metabolic process (GO:0035337, cluster 1, IC = 7.97) with two genes down-regulated at EID11 (ELOVL7 and DGAT2), fatty acid catabolic process (GO:0009062, cluster 3, IC = 6.96) with three genes down-regulated at EID11 (HACL1, AIG1, ACOX3), protein lipidation (GO:0006497, cluster 4, IC = 6.61) with five genes up-regulated at EID11 and EID15 (ATG12, UGCG, ZDHHC21, ATG10 and PIK3C3) of which four are located on chromosome Z, and lipoprotein biosynthetic process (GO:0042158, cluster 4, IC = 6.58) with five genes up-regulated at EID11 and EID15 (ATG12, UGCG, ZDHHC21, ATG10 and PIK3C3). A high value of IC indicates that the GO terms are specific.

The analysis of cluster 9 (dark green color) also revealed two GO terms related to sex determination (GO:0007530 and GO:0030238) with two unique genes that are down-regulated at EID11 (DMRT1, located on Z chromosome, and FGF9), and two GO terms related to bone formation and morphogenesis (GO:0060346 and GO:0061430) including three unique genes up-regulated at EID15 (MMP2, SEMA4D and FBN2).

This functional data (Table S3 and Fig. 4) can be explored by the entries of differentially expressed genes, enriched GO terms or cluster of GO terms, giving the user many possibilities to interpret the data.

## 4 Experimental Design, Materials and Methods

The experimental procedure was previously described [1] and is briefly summarized in the next paragraphs.

### 4.1 Egg handling and extraction of CAM RNA

Fertilized eggs laid by 33-week-old broiler hens (Ross 308) were purchased from a French hatchery (Boyé Accouvage, La Boissière en Gâtines, France) and handled in the Poultry Experimental Facility (PEAT) UE1295 (INRAE, F-37380 Nouzilly, France, DOI:10.15454/1.5572326250887292E12). Eggs were stored for three days at 75 % RH, 16 °C and then incubated for 11 or 15 days under standard conditions (45 % RH, 37.8 °C, automatic turning every hour, Bekoto B64-S, Pont-Saint-Martin, France). For each embryonic day studied (after either 11 or 15 days, EID11 or EID15), the CAM was carefully removed from the eggshell, rinsed with sterile saline solution, and immediately frozen in liquid nitrogen prior to storage at – 80 °C.

All CAM samples (10 male CAMs and 10 female CAMs per EID) were independently homogenized in liquid nitrogen with a mechanical crusher A11 Basic (IKA, Staufen im Breisgau, Germany). RNA extraction was achieved using the Nucleospin RNA kit (Macherey-Nagel, Düren, Germany), followed by a treatment with DNAse (kit Turbo DNA-freeTM, Life Technologies, Carlsbad, USA) to remove any trace of genomic DNA. The sample quality was assessed by measuring 260 nm /280 nm (ND 1000 spectrophotometer, Thermoscientific, Waltham, MA, USA) to evaluate RNA purity and after migration on a 1 % agarose gel. For samples dedicated to RNA sequencing, RNA integrity was checked using Agilent 2100 bioanalyzer (Santa Clara, CA, USA).

### 4.2 Sequencing libraries and quantification

The library preparations were sequenced on an Illumina platform (Illumina NovaSeq 6000 S4 flowcell with PE150, Novogene, Cambridge, CA, UK) and paired-end reads were generated. The kit was NEB Next® Ultra™ RNA Library Prep Kit. Raw data (raw reads) in FASTQ format were cleaned by removing reads containing adapter, reads containing ploy-N and low quality reads from raw data. At the same time, Q20, Q30 and GC content were calculated. All the downstream analyses were based on the clean data with high quality.

Reference Gallus gallus genome (GRCg6a (GCA_000002315.5)) and gene model annotation files were downloaded from genome website directly. Index of the reference genome was built and paired-end clean reads were aligned to the reference genome using HISAT2 v2.0.5 software [8] (with command hisat2 -x hisat2-idx -p 4 –dta -t –phred33 -1 sample_1.clean.fq.gz -2 sample_2.clean.fq.gz –un-conc-gz sample.unmap.fq.gz 2>sample_align.log|samtools sort -O BAM –threads 4 -o sample.bam). The mapped reads of each sample were assembled by StringTie (v1.3.3b) [9] in a reference-based approach. StringTie uses a novel network flow algorithm as well as an optional de novo assembly step to assemble and quantitate full-length transcripts representing multiple splice variants for each gene locus.

Quantification HTSeq v0.6.1 [10] (with default parameters and the union mode) was used to count the read numbers mapped of each known and novel genes.

### 4.3 Differential analysis

All statistical analyses were performed with packages using R software v.4.2.1 [11] and Bioconductor project v.3.17. In the multidimensional plot explaining 56 % of the gene expression variability, all samples are grouped by biological conditions except for EID15 female sample number 83, which was removed from the subsequent statistical analyses (Fig. 1). A filtering step was applied to retain genes with a count per million (cpm) greater than 1 in at least 9 samples (the minimum number of biological replicates). The counts of the 15,124 expressed genes on the total of 26,151 genes of the 39 sequencing librairies were normalized by trimmed mean of M values (TMM) from edgeR R package (version 3.38.4) [12] taking into account the distribution of reads. For each gene, a negative binomial generalized linear model (GLM) [13] was fitted with a group factor combining the factors “day of incubation” (EID11, EID15) and “sex”. P-values were adjusted by controlling the false discovery rate (<0.05) using Benjamini-Hochberg correction [14]. Genes differentially expressed between male and female CAM [2] were obtained for each incubation stage using the likelihood ratio test on the two defined contrasts EID11M – EID11F and EID15M – EID15F (Table S1 and Table S2, respectively). The results of the differential analyses were visualized using the Volcano plot (Fig. 2), a scatterplot showing statistical significance (adjusted P-values) versus fold change (log2FC) using the EnhancedVolcano package (version 1.18.0).

### 4.4 Functional enrichment test

The relationships between genes and biological functions were explored based on gene ontology (GO) covering biological process using ViSEAGO Bioconductor package (version 1.17.0) [15]. Significantly enriched biological process GO terms were obtained using a classical Fisher test with a significance threshold of 0.01 with GO annotation using the Ensembl Genes 106 database. Enriched GO terms were clustered using a hierarchical clustering algorithm based on the Wang's distance between GO terms and the ward.D2 aggregation criteria. Clusters with similar semantic similarity between the GO terms were identified after dynamic cutting of the dendrogram (Table S3, Fig. 4). This new dataset containing functional information on sex-linked genes in CAM at 11 and 15 days of incubation can be interactively explored and analyzed.

## Limitations

We used eggs from a meat-strain chicken strain that were selected for decades on their growth performance. Developmental stages are expected to be essentially comparable regardless of the genetic origin of chickens but may slightly differ between strains and depending on the conditions used for egg storage and incubation. CAM samples used in this experiment were collected on the equatorial region of the egg where the CAM is in contact with the eggshell. It is hypothesized that the transcriptomes of the CAM collected on the large end of the egg (in contact with the air cell) and the blunt end (in contact with egg white) are different from the one presented in this dataset.

## Ethics Statement

All experiments using fertilized eggs comply with the ARRIVE guidelines [16]. They followed the European legislation on the “Protection of Animals Used for Experimental and Other Scientific Purposes” (2010/63/UE), and the guidelines approved by the institutional animal care and use committee (IACUC).

## Credit Author Statement

**Christelle Hennequet-Antier**: Software, Methodology, Validation, Formal Analysis, Data Curation, Writing - Original Draft, Visualization. **Maeva Halgrain**: Data curation, Investigation, Writing - Review & Editing. **Sophie Réhault-Godbert:** Conceptualization, Supervision, Writing - Original Draft, Review & Editing.

## Data Accessibility

The datasets supporting the results and the discussion are available at the Gene Expression Omnibus (GEO) repository at http://www.ncbi.nlm.nih.gov/geo/ using the accession number GSE199780. The script R and the results for the differential analysis and the functional enrichment tests (supplementary files Tables S1-S3) were stored in the repository Hennequet-Antier, Christelle, 2024, “CAM_RNA-Seq”, https://doi.org/10.57745/6YDAQD, Recherche Data Gouv.

## Data Availability

Transcriptome of the chicken chorioallantoic membrane from male and female embryos, after 11 and 15 days of incubation (Original data) (Gene Expression Omnibus (GEO)).

## Appendix Supplementary materials

Image, application 1

Image, application 2

Image, application 3

Image, application 4

## References

[1] Halgrain M., Bernardet N., Hennequet-Antier C., Hincke M., Réhault-Godbert S. (2023). RNA-seq analysis of the active chick embryo chorioallantoic membrane reveals genes that encode proteins assigned to ion transport and innate immunity. Genomics.

[2] Halgrain M., Bernardet N., Hennequet-Antier C. (2024). Réhault-Godbert S. Sex-specific transcriptome of the chicken chorioallantoic membrane. Genomics.

[3] Makanya A.N., Dimova I., Koller T., Styp-Rekowska B., Djonov V. (2016). Dynamics of the developing chick chorioallantoic membrane assessed by stereology, allometry, immunohistochemistry and molecular analysis. PLoS ONE.

[4] Ahmed T.A.E., Cordeiro C.M.M., Elebute O., Hincke M.T. (2022). Proteomic analysis of chicken chorioallantoic membrane (CAM) during embryonic development provides functional insight. BioMed Res. Int..

[5] Gabrielli M.G., Accili D. (2010). The chick chorioallantoic membrane: a model of molecular, structural, and functional adaptation to transepithelial ion transport and barrier function during embryonic development. J. Biomed. Biotechnol..

[6] Ribatti D. (2017). The chick embryo chorioallantoic membrane (CAM) assay. Reprod. Toxicol*.*.

[7] Degalez F., Charles M., Foissac S., Zhou H., Guan D., Fang L., Klopp C., Allain C., Lagoutte L., Lecerf F., Acloque H., Giuffra E., Pitel F., Lagarrigue S. (2024). Enriched atlas of lncRNA and protein-coding genes for the GRCg7b chicken assembly and its functional annotation across 47 tissues. Sci. Rep..

[8] Kim D., Paggi J.M., Park C., Bennett C., Alzberg S.L.S (2019). Graph-based genome alignment and genotyping with HISAT2 and HISAT-genotype. Nat. Biotechnol..

[9] Pertea M., Pertea G.M., Antonescu C.M., Chang T.C., Mendell J.T., Salzberg S.L. (2015). StringTie enables improved reconstruction of a transcriptome from RNA-seq reads. Nat. Biotechnol..

[10] Putri G.H., Anders S., Pyl P.T., Pimanda J.E., Zanini F. (2022). Analysing high-throughput sequencing data in Python with HTSeq 2.0. Bioinformatics.

[11] R Core Team. A Language and Environment for Statistical Computing, R Foundation for Statistical Computing*.*

[12] Robinson M.D., McCarthy D.J., Smyth G.K. (2010). EdgeR: a Bioconductor package for differential expression analysis of digital gene expression data. Bioinformatics.

[13] McCarthy D.J., Chen Y., Smyth G.K. (2012). Differential expression analysis of multifactor RNA-Seq experiments with respect to biological variation?. Nucleic Acids Res..

[14] Benjamini Y., Hochberg Y. (1995). Controlling the false discovery rate: a practical and powerful approach to multiple testing. J. R. Stat.Soc. Ser. B (Methodol.).

[15] Brionne A., Juanchich A., Hennequet-Antier C. (2019). ViSEAGO: a bioconductor package for clustering biological functions using gene ontology and semantic similarity. BioData Min..

[16] Percie du Sert N., Hurst V., Ahluwalia A., Alam S., Avey M.T., Baker M. (2020). The ARRIVE guidelines 2.0: updated guidelines for reporting animal research. PLOS Biol..

