# Supplementary material for: RNA-seq dataset of the chorioallantoic membrane of male and female chicken embryos, after 11 and 15 days of incubation
Source: Data Brief. 2024 Aug 14;56:110830. doi: 10.1016/j.dib.2024.110830 (PMC11388263; doi:10.1016/j.dib.2024.110830)
Supplement: Supplementary file 4 [file mmc4.docx]

# APPENDIX. Supplementary materials

These tables were available from the dataset Hennequet-Antier, Christelle, 2024, "CAM_RNA-Seq", <https://doi.org/10.57745/6YDAQD> in the Recherche Data Gouv repository.

**Table S1: EID11_MvsF.xlsx**

This file contains the results of the differential analysis performed between male and female CAMs at EID11 using edge R package. The first sheet, named “complete”, contains the results on the genes after a filtering step based on the count per million criteria. The first five columns provide a description of the gene from the GRCg6a Gallus gallus genome reference, followed by the counts and the normalized counts using TMM for the 39 samples. Average counts were calculated for all samples and by group. A likelihood ratio test between male and female of EID11 CAMs was performed using a negative binomial generalized linear model (GLM) with a group factor combining the factors “day of incubation” (EID11, EID15) and “sex”. Parameters of the statistical model were estimated and reported in the table: Fold Change (FC) and its transformation into log2 (log2FoldChange), pvalue of the statistical test and its Benjamini-Hochberg’s correction (padj), the estimated dispersion tagwise and trended.

The "up" and "down" sheets are extracted from the "complete" table and contain genes that are significantly up-regulated (FC >0 and padj < 0.05), i.e. gene expression is higher in males than in females, and genes that are significantly down-regulated (FC <0 and padj < 0.05), i.e. gene expression is higher in females than in males.

**Table S2: EID15_MvsF.xlsx**

This file contains the results of the differential analysis performed between male and female of EID15 CAMs using edge R package. Presentation is the same as for Table S1.

**Table S3: GOcluster_heatmap_Wang_wardD2.xls**

The functional enrichment test of the four lists of differentially expressed genes (EID11_MvsF_down, EID15_MvsF_down, EID11_MvsF_up, EID15_MvsF_up) was performed using ViSEAGO Bioconductor package. The hierarchical clustering algorithm was built from the enriched GO terms with the Wang’s distance and the ward.D2 aggregation criteria. Then, the enriched GO terms were grouped by cluster by cutting the dendrogram. The resulting file includes, for each enriched GO term, the cluster number, and the information content (IC) which is computed as the negative log probability of occurrence of the term within all GO term. For each list of differentially expressed genes, the file also contains the frequency of differentially expressed genes among all genes in the cluster, the p-value of the enrichment test and the differentially expressed genes.
